# Short-term efficacy and tolerability of venlafaxine extended release in adults with generalized anxiety disorder without depression: A meta-analysis

**DOI:** 10.1371/journal.pone.0185865

**Published:** 2017-10-05

**Authors:** Xinyuan Li, Lijun Zhu, Yingying Su, Shaokuan Fang

**Affiliations:** 1 Department of Neurology, Neuroscience Centre, the First Teaching Hospital of Jilin University, Changchun, China; 2 China-Japan Union Hospital of Jilin University, Changchun, China; 3 Department of Epidemiology and Biostatistics, School of Public Health of Jilin University, Changchun, China; University of Catanzaro, ITALY

## Abstract

**Background:**

Although efficacy of venlafaxine extended release (XR) for generalized anxiety disorder (GAD) has been reported in previous analyses in 2002 and 2004, the sample size was rather small and estimate of safety or tolerability was not clear. The present analysis had the advantage of large sample size and provided evidence for tolerability.

**Methods:**

Literature databases were searched, including Pubmed, Embase, Cochrane Central Register of Controlled Trials, Web of science and clinical trials. 10 eligible articles were finally selected and data was extracted and logged into the Review Manager 5.3 by two independent authors. The risk of bias was evaluated by the Cochrane Collaboration’s Risk of Bias Tool and the stability of the results was assessed by sensitivity analysis. The publication bias was assessed by funnel plot and Egger’s/Begg’s test using Stata Version 12.0 software.

**Results:**

In the current meta-analysis, 10 articles (14 studies) satisfying the inclusion criteria were analyzed. As efficacy outcomes, our findings indicated venlafaxine XR was significantly more effective than placebo according to mean change of the Hamilton Rating Scale for Anxiety total scores [mean difference = 3.31, 95% confidence interval(CI) 1.44–5.18, P = 0.0005], response [odds ratio(OR) = 1.83, 95%CI 1.58–2.12, P<0.00001], and remission (OR = 2.55, 95%CI 1.36–4.78, P = 0.003). In terms of tolerability, the most frequently reported treatment-emergent adverse events were nausea, dry mouth, dizziness, insomnia, somnolence, and headache. In addition, discontinuation due to all-cause (OR = 1.17, 95%CI 0.92–1.49, P = 0.19) was not significantly different between the two groups, whereas discontinuation due to adverse events was statistically higher in the venlafaxine XR group compared with the placebo treatment (OR = 2.80, 95%CI 2.21–3.54, P<0.00001) and discontinuation due to inefficacy was lower in venlafaxine than placebo treatment (OR = 0.26, 95%CI 0.17–0.40, P<0.00001). There was no significant publication bias and sensitivity analysis showed that our analysis exhibited high stability.

**Conclusion:**

We concluded that venlafaxine XR (75–225 mg/day) is an effective and well-tolerated pharmacological treatment option for adult patients with GAD.

## Introduction

Generalized anxiety disorder (GAD) is a common disorder with an estimated lifetime prevalence of 4.3–5.9%[1]. GAD is typically diagnosed when excessive anxiety in association with routine events (e.g., work, relationships, health) occurs on most days for a duration of at least 6 months, accompanied by somatic and psychic complaints such as restlessness, irritability, muscle tension, fatigue, difficulty concentrating, and sleep disturbance [2]. Patients with GAD are often prompted to seek treatment and primary care due to these accompanying complaints. In addition, patients with GAD present higher rates of comorbid illnesses such as major depressive disorder, bipolar disorder, cardiovascular disease [3], diabetes, and arthritis[4].

Currently, the treatment of GAD involves several classes of medications. Antidepressants such as the selective serotonin reuptake inhibitors(SSRIs) and selective norepinephrine/noradrenaline reuptake inhibitors (SNRIs) are generally regarded as first-line medications[5]. In addition, tricyclic antidepressants, buspirone, benzodiazepines, anticonvulsants, and pregabalin are also alternative interventions for GAD treatment[2]. The selection of different pharmacological agents is mainly based on their efficacy and adverse events (AEs). The response and remission based on the total scores of the Hamilton Rating Scale for Anxiety (HAM-A) and Clinical Global Impression are common standards used to evaluate the efficacy. AEs mainly included nausea, dry mouth, dizziness, insomnia and so on.

Venlafaxine extended release (XR), which has been approved by the United States Food and Drug Administration (FDA) in 1993[6], is a SNRI that increases the synaptic levels of serotonin and noradrenaline by preventing their reuptake through binding to monoamine transporter sites. Thus, venlafaxine XR is used for the treatment of GAD [7]. Randomized controlled trials (RCTs) have also demonstrated a higher efficacy of venlafaxine XR in treating GAD compared with other medications such as duloxetine, pregabalin, and benzodiazepines[8–10].

Katz et al[11] published a pooled analysis of venlafaxine XR in older adults with GAD, including three 8-week and two 24-week RCTs, to evaluate its short- and long-term efficacy. Subsequently, Meoni et al[12] analyzed the efficacy of venlafaxine XR on somatic and psychic symptoms in patients with GAD using the same five studies and found patients treated with venlafaxine XR showed similar somatic and psychic anxiety response rates while patients receiving placebo had higher somatic compared with psychic response rate. The obvious limitations in both studies were the rather small sample size and unclear estimate of the safety or tolerability.

A number of RCTs have been conducted on the efficacy and tolerabolity of venlafaxine in GAD, but their results have been inconsistent. Therefore, we systematically reviewed all published and non-published RCTs on venlafaxine XR in adults with GAD to determine its short-term efficacy and tolerability.

## Methods

### Search strategy

The relevant studies published until April 4, 2017 were searched using PubMed, Embase, web of science, Cochrane Central Register of Controlled Trials (CENTRAL) hosted by the Cochrane Library, and registry of clinical trials (www.clinicaltrials.gov) according to the Preferred Reporting Items for Systematic Review and Meta-Analyses (PRISMA) working group (S1 File)[13]. We followed a detailed methodology described in the protocol (S2 File). The search terms were (“Venlafaxine” OR “Effexor” OR “Efexor” OR “Vandral” OR “Trevilor” AND “Generalized Anxiety Disorder” OR “Anxiety”). Moreover, Medical Subject Headings (MeSH) or keywords were used when available. In order to avoid missing important studies, trials were further identified in the reference lists of narrative reviews. An additional search was conducted on May 9, 2017 using the same search engines. There were no restrictions related to the language or date of publication.

### Inclusion and exclusion criteria

Studies meeting the following inclusion criteria were selected: (1) patients aged or older than 18 years meeting the Diagnosis and Statistical Manual of Mental Disorders, Fourth Edition (DSM-IV)[14]criteria for GAD; (2) venlafaxine XR or venlafaxine XR plus antipsychotics lasting ≤10 weeks; (3) placebo-controlled; (4) efficacy and tolerability data; and (5) RCTs. We excluded studies that met the following criteria: (1) DSM-IV diagnosis of major depressive disorder, bipolar disorder, or other psychotic disorders within the previous 6 months; (2) use of any neuroleptic, antidepressant, or anxiolytic medication within 2 weeks of the baseline visit (5 weeks for fluoxetine and 30 days for benzodiazepine); (3) history of alcohol or psychoactive substance abuse or dependence within the past 6 months; (4)patients at risk of suicide; (5) previous treatment with venlafaxine before randomization; (6) treatment outcomes not available.

### Data extraction

Two authors (Xinyuan Li and Lijun Zhu) independently assessed the quality of the selected studies and extracted the data using data extraction forms. Disagreements were resolved by reaching a consensus through a third author. The extracted data mainly included age, sex distribution, number of enrolled participants, study-level inclusion and exclusion criteria, intervention details, treatment duration, reported outcomes, efficacy measures, and measure times. When multiple measures were used, HAM-A was the first choice for data extraction.

### Outcomes and definitions

For the efficacy analysis, the last-observation-carried-forward (LOCF) approach was applied. The primary efficacy parameter was the mean change in HAM-A total scores from baseline to endpoint[15]. The secondary efficacy parameters were the response and remission rates. The response was defined as ≥50% reduction from baseline in the HAM-A total score, while the remission corresponded to HAMA total score ≤7 at endpoint[16]. With regard to tolerability endpoints for the analysis, the primary tolerability outcomes were rates of discontinuations due to all-cause, AEs, and lack of efficacy, common TEAEs in the venlafaxine XR treatment was assessed as secondary tolerability outcomes.

### Quality assessment

Two authors (Xinyuan Li and Yingying Su) independently assessed the risk of bias and disagreements were resolved by discussion in the research team. According to the Cochrane Collaboration’s Risk of Bias Tool[17], the likelihood of risk of bias included the selection bias (random sequence generation, allocation concealment), detection bias (blinding of outcome assessors, participant/personnel), reporting bias (selective reporting), and attrition bias (incomplete outcome data).

### Statistical analysis

The primary and secondary efficacies as well as the discontinuation rates were measured using intent-to-treat (ITT) analysis. Data evaluating the primary efficacy was regarded as continuous data. We presented the effect size (ES) as the mean difference (MD) using an analysis of covariance (ANCOVA) model (Hedges’ g data) with 95% confidence interval (CI). Therefore, the mean and standard deviation (SD) were calculated for each selected study.

**Fig 1:**
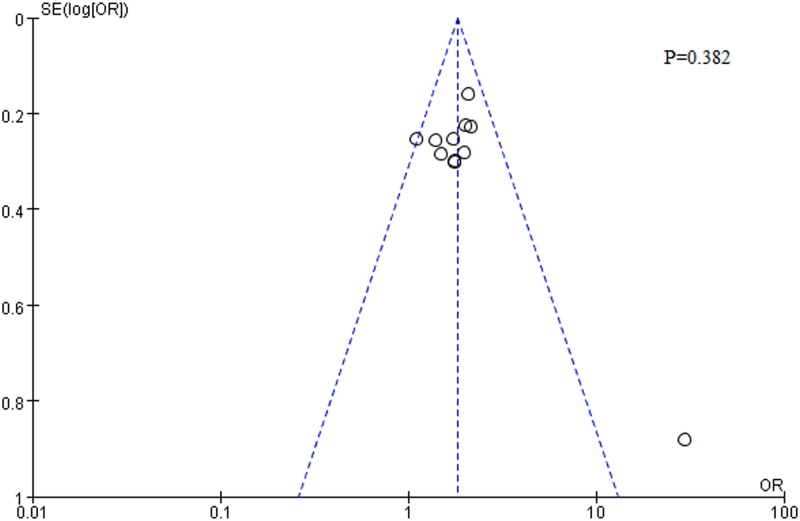
Funnel plot of publication bias.

Dichotomous data, response, remission, and discontinuation rates at endpoint were analyzed using the Mantel-Haenszel (M-H) fixed-effects or random-effects models. Study heterogeneity was evaluated using the I^2^ statistic; a value of 0% indicated no heterogeneity, 50% indicated moderate heterogeneity, and 75% indicated high heterogeneity. In general, heterogeneity was defined as P<0.05 and I^2^≥50%[18–19]. The ES was presented as odds ratio (OR) with corresponding 95%CI. For the final analysis, all extracted data were entered into the Review Manager 5.3 software provided by the Cochrane Collaboration (London, UK). The significance of the pooled estimates was determined by Z statistic, a statistical significance was set at a two-tailed P<0.05. The publication bias was assessed by the funnel plot and the Begg’s/Egger’s test[20–21]using Stata Version 12.0 software and there was no publication bias(P = 0.382)(Fig 1). If substantial heterogeneity was identified, the sensitivity analysis was performed. When we converted fixed effect model to random effect model in heterogeneity outcomes, the pooled ORs were all located in the significant range of overall effect, indicating that the results of the meta-analysis showed low sensitivity and high stability[22].

## Results

### Characteristics of included studies

**Fig 2:**
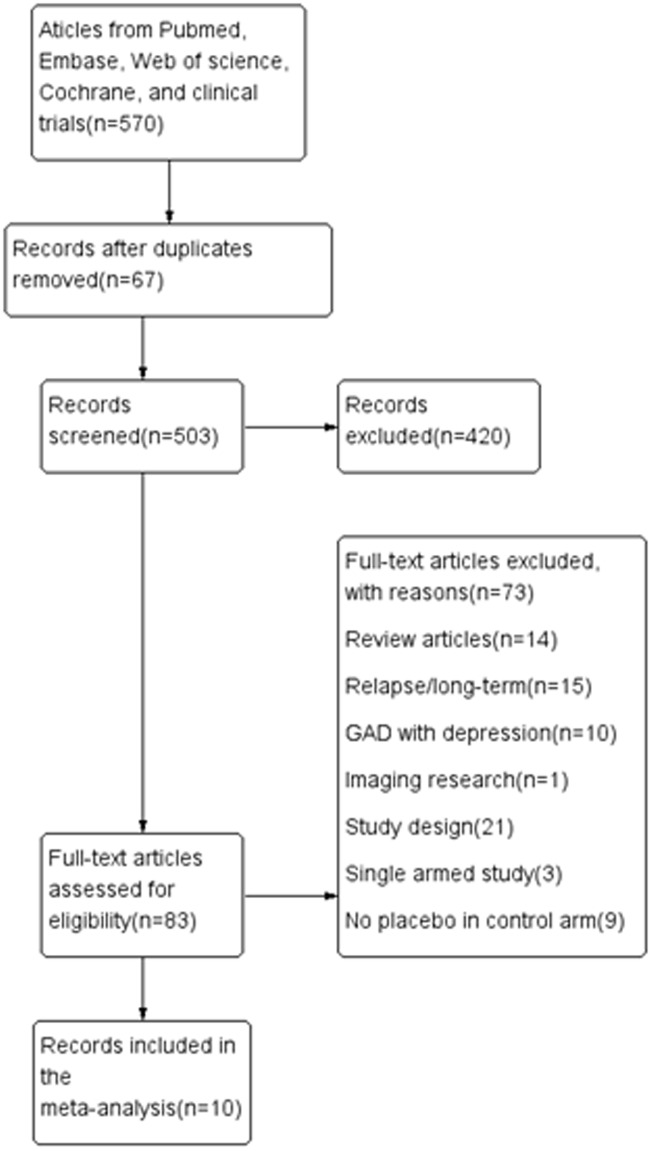
Flow diagram of the literature search.

Fig 2 illustrated the flowchart of the inclusion process and exclusion criteria. A total of 570 articles were initially retrieved from Pubmed, Embase, Cochrane database, Web of science or clinical trials. We excluded in total 420 irrelevant articles, 67 duplicate papers based on the title or abstract review, and 83 articles after full-text reading. Finally, 10 eligible articles (14 studies) were included in the present meta-analysis. The meta-analysis further included a combined sample of 3,622 patients with moderately severe GAD from 14 short-term RCTs comparing venlafaxine XR (1,883 patients) with placebo (1,739 patients) that fulfilled the eligibility criteria. All enrolled studies were conducted between 1999 and 2009. We summarized the main feature of these 14 short-term RCTs in Table 1. Ten studies lasted for 8 weeks, three lasted for 10 weeks, and one lasted for 6 weeks. There was no restriction regarding the fixed or flexible administered doses. Six studies involved flexible doses of venlafaxine XR (75–225 mg/day), the others included single fixed doses of venlafaxine XR (75, 150, or 225 mg/day).

**Table 1:** Characteristics of multicentres, randomized, double-blind, placebo-controlled studies included in the meta-analysis.

Study	Treatment/control	VXRDose(mg/d)	Duration(weeks)	Mean age years(±SD)	Sex(F, %)	PatientPopulation	Sample Size(N^a^)	Baseline HAM-A mean(±SD)	Entry score^b^	Mean change of HAM-A(±SD)^c^	Response(%)^d^	Remission(%)^e^	Location
**Davidson 1999a**[23]	VXR/PBO	75	8	38±10/39±11	59.8/62.2	GAD(DSM-IV)	87/98	23.7±4.1/23.7±4.2	≥18	-	49/36	-	The US
**Davidson 1999b**[23]	VXR/PBO	150	8	37±11/39±11	69.0/62.2	GAD(DSM-IV)	87/98	23.0±4.0/23.7±4.2	≥18	-	49/36	-	the US
**Rickels 2000a**[24]	VXR/PBO	75	8	40.4±12.8/40.9±11.3	58/57	GAD (DSM-IV)	86/96	24.7±4.4/24.1±4.2	≥18	11.22/9.51	-	-	the US
**Rickels 2000b**[24]	VXR/PBO	150	8	39.6±11.9/40.9±11.3	56/57	GAD(DSM-IV)	81/96	24.5±4.1/24.1±4.2	≥18	12.36/9.51	-	-	the US
**Rickels 2000c**[24]	VXR/PBO	225	8	42.4±12.3/40.9±11.3	52/57	GAD (DSM-IV)	86/96	23.6±3.7/24.1±4.2	≥18	11.52/9.51	-	-	the US
**Hackett 2003a**[10]	VXR/PBO	75	8	45/43	67/64	GAD (DSM-IV)	191/97	27.9/27.6	≥20	14.0/11.7	59/45	-	Helsinki
**Hackett 2003b**[10]	VXR/PBO	150	8	44/43	66/64	GAD (DSM-IV)	179/97	27.9/27.6	≥20	12.8/11.7	54/45	-	Helsinki
**Nimatoudis 2004**[25]	VXR/PBO	75–150	8	41±1444±12	66.7/68.2	GAD (DSM-IV)	24/22	27.1±4.8/28.5±6.4	>18	19.2±5.1/10.8±8.1	92/27	62.5/9.1	Greece
**Montgomery 2006**[9]	VXR/PBO	75	6	46±12/43±12	65/58	GAD (DSM-IV)	113/101	26.0±4.6/27.4±5.5	≥20	14.1±8.4/11.6±8.0	62/45	-	5 European Countries
**Hartford 2007**[26]	VXR/PBO	75–225	10	40.1±13.2/41.9±14.2	62.2/61.5	GAD (DSM-IV)	164/161	24.9±5.4/25.0±5.8	-	12.4±8.6/9.19±8.5	54/37	30/19	the US
**Bose 2008**[27]	VXR/PBO	75–225	8	37.1±10.8/37.6±12.3	59.7/62.5	GAD (DSM-IV)	133/140	23.8±3.5/23.7±3.5	≥20	-	52/42.2	31.2/23.7	the US
**Allgulander 2008**[8]	VXR/PBO	75–225	10	41.6±13.2	60.6	GAD (DSM-IV-TR)	333/331	26.1±6.7/26.2±6.7	-	-	58/40	-	the US
**Kasper 2009**[4]	VXR/PBO	75–225	8	42.6±11.8/40.2±12.1	58/61	GAD (DSM-IV-TR)	125/128	27.4±4.5/26.8±9.1	≥20	12.0±10.1/11.7±10.2	46/44	-	8 countries
**Nicolini 2009**[28]	VXR/PBO	75–225	10	42.8	57.1	GAD (DSM-IV)	169/170	27.4±7.6/27.3±7.3	-	15.5±8.8/11.6±8.9	61/42	44/20	8 countries

VXR, Venlafaxine XR; PBO, Placebo; SD, standard deviation; HAM-A, Hamilton anxiety rating scale; LOCF, Last observation carried forward; -, not applicable.

^a^ Number treated.

^b^ By HAM-A total score.

^c^ Primary end-point measure(LOCF).

^d^ Response defined as≥50% reduction in HAM-A total score at the endpoint(LOCF).

^e^ Remission defined as HAM-A≤7 at the endpoint(LOCF).

### Quality assessment

**Fig 3:**
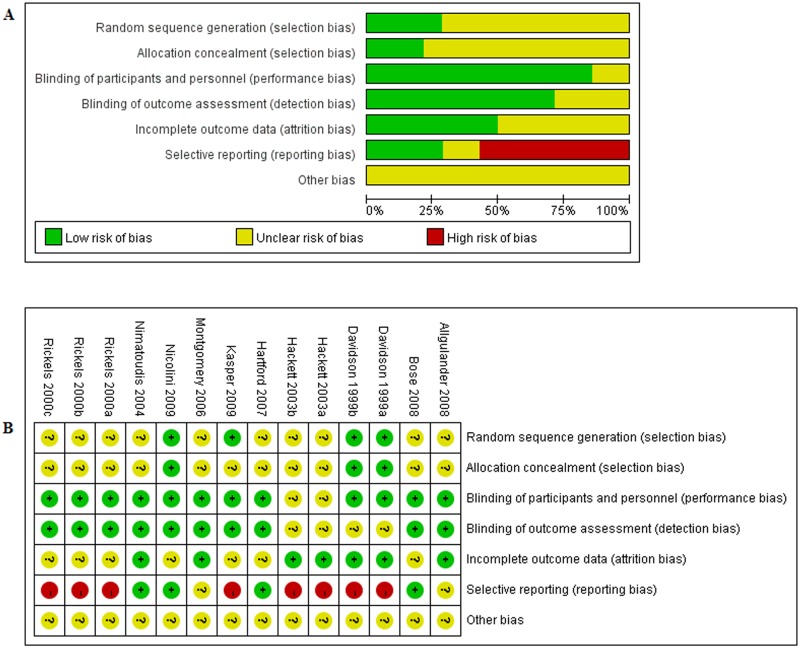
A risk of bias gragh, B risk of bias summary(“+”low risk;“?”, unclear risk;“-”,high risk).

The quality of study was assessed by the Cochrane Collaboration’s Risk of Bias Tool[17], seen in Fig 3, risk of bias across studies was shown in Fig 3A and risk of bias in individual studies was shown in Fig 3B

### Outcomes

#### Primary efficacy outcome

**Fig 4:**
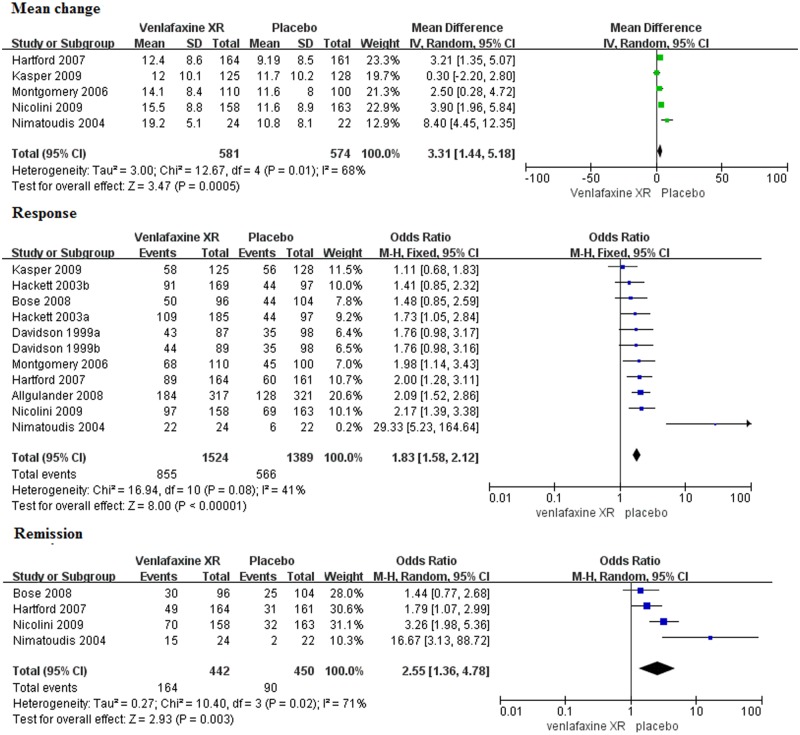
Forest plots of primary and secondary efficacy outcomes. SD, standard deviation; CI, confidence interval; M-H, Mantel-Haenszel.

The primary efficacy at endpoint (LOCF) was shown as a forest plot (Fig 4). Five studies with a total of 1,155 patients were included in the meta-analysis of the mean change from baseline to endpoint on the HAM-A total scores. Nine studies were excluded since five provided no information for the SD or standard error (SE), while the other four did not report the calculating mean change from baseline. The results indicated a significantly larger reduction of the HAM-A total scores in venlafaxine XR than placebo group (MD = 3.31, 95%CI 1.44–5.18, P = 0.0005), and heterogeneity was detected (I^2^ = 68%, P = 0.01), thus, a random-effects model was used.

#### Secondary efficacy outcomes

Eleven studies reported the response based on the HAM-A total score and a combined sample of 2,913 patients was included in the response analysis (Fig 4). The OR for venlafaxine XR (OR = 1.83, 95%CI 1.58–2.12, P<0.00001) indicated that patients treated with venlafaxine XR responded more than those treated with placebo; heterogeneity was not detected (I^2^ = 41%, P = 0.08). Four studies reported remission based on the HAM-A total score and a combined sample of 892 patients was included in the remission analysis (Fig 4). The OR in the venlafaxine XR group (OR = 2.55, 95%CI 1.36–4.78, P = 0.003) indicated a significant difference compared with the placebo group. In this case, heterogeneity was detected (I^2^ = 71%, P = 0.02), thus, a random-effects model was used.

#### Primary tolerability outcomes

**Fig 5:**
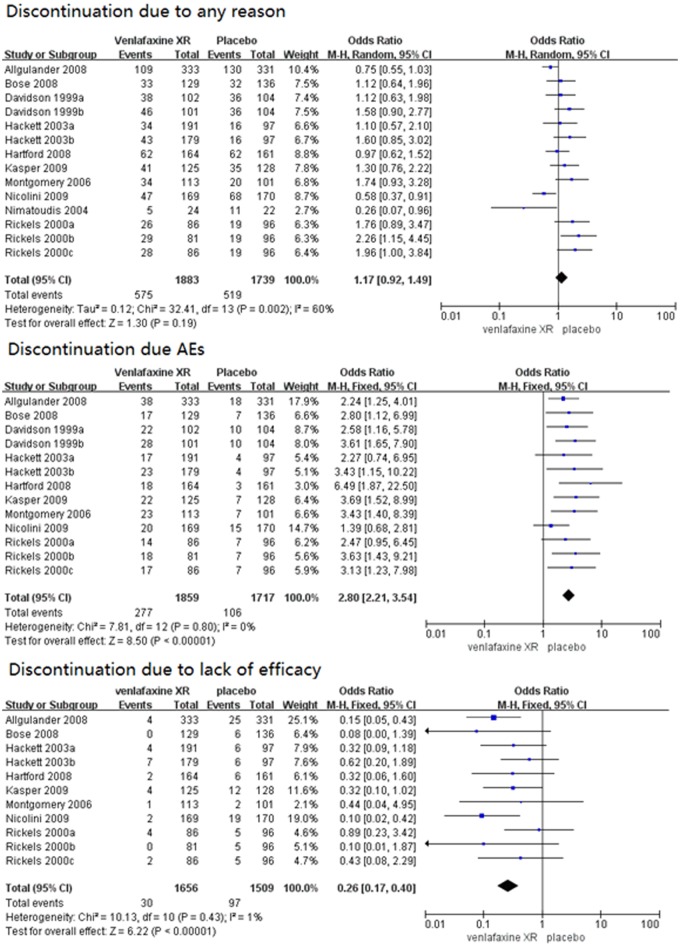
Forest plots of discontinuation due to any reason, AEs, and lack of efficacy. AEs, adverse effects; SD, standard deviation; CI, confidence interval; M-H, Mantel-Haenszel.

The tolerability of venlafaxine XR was evaluated by the discontinuation rates due to any reason, AEs, and lack of efficacy(Fig 5). No significant difference was observed between the venlafaxine XR and placebo groups regarding the discontinuation for any reason (OR = 1.17, 95%CI 0.92–1.49, P = 0.19). Heterogeneity was detected (I^2^ = 60%, P = 0.002), thus, a random-effects model was used, whereas the discontinuation rate due to AEs in the venlafaxine XR group was significantly higher than the placebo group (OR = 2.80, 95%CI 2.21–3.54, P<0.00001) In this case, no heterogeneity was detected (I^2^ = 0%, P = 0.80). Nine studies were included because one study[25] did not provide information about the discontinuation rates due to AEs. The discontinuation rates due to lack of efficacy in the venlafaxine XR group were significantly lower than the placebo group (OR = 0.26, 95%CI 0.17–0.40, P<0.00001). In this case, no heterogeneity was found (I^2^ = 1%, P = 0.43). Eleven studies were included, while the remaining three[23,25] were excluded because they did not report the lack of efficacy.

#### Secondary tolerability outcomes

The incidence of overall TEAEs was not extracted bacause almost all studies did not report clearly. However, among all the included studies, the most frequently reported TEAEs were nausea, dry mouth, dizziness, insomnia,somnolence, and headache(Table 2). Nausea, reported most commonly in the venlafaxine XR group, was experienced ranging from mild to moderate and occurred early during the course of the treatment[8]. The incidence of nausea, dry mouth, dizziness, insomnia, and somnolence was all significantly higher in the venlafaxine XR than in the placebo group. It is worth mentioning that no significant difference was observed in headache between the two groups (OR = 1.00, 95%CI 0.66–1.54, P = 0.98). In addition, three studies reported blood pressure variations. Nimatoudis et al[25] reported no significant mean changes in blood pressure (systolic with baseline 128.1mmHg and last 124.0mmHg; diastolic with baseline 82.3mmHg and last 79.0mmHg), consistent with the other two studies. Moreover, mean changes from baseline to endpoint in blood pressure were not significantly different between venlafaxine XR and placebo group with systolic pressure(OR = -0.62, 95%CI -2.38,1.14[8]; OR = 2.26, 95%CI -0.46,4.98[27])and diastolic pressure(OR = 0.34, 95%CI -1.05–1.73[8]; OR = 2.26, 95%CI 0.34–4.18[27]).

**Table 2:** Meta-analysis of most frequent TEAEs.

TEAEs	Included Studies(N)	OR	heterogeneity	Effect Model	Merger value	95%CI
**Nausea**	10[4,9,23–27]	4.07	P = 0.22, I^2^ = 24%	Fixed	P<0.00001	3.23–5.14
**Dry mouth**	10[4,9,23–27]	4.19	P = 0.97, I^2^ = 0%	Fixed	P<0.00001	3.05–5.76
**Dizziness**	7[4,9,23–24]	1.91	P = 0.94, I^2^ = 0%	Fixed	P<0.0001	1.40–2.60
**Insomnia**	7[4,9,24,26–27]	2.03	P = 0.42, I^2^ = 0%	Fixed	P<0.00001	1.49–2.77
**Somnolence**	7[4,9,24,26–27]	2.36	P = 0.70, I^2^ = 0%	Fixed	P<0.00001	1.68–3.31
**Headache**	3[4,9,27]	1.00	P = 0.39, I^2^ = 0%	Fixed	P = 0.98	0.66–1.54

TEAEs, treatment-emergent adverse events; OR, odds ratio; CI, confidence interval.

## Discussion

To the best of our knowledge, this is the largest comprehensive meta-analysis for the efficacy and tolerability of venlafaxine XR as an anti-anxiety medication for the short-term treatment of GAD. Although our some efficacy findings were consistent with previous meta-analyses[11,12], the present analysis had the advantage of the large sample size, which provided sufficient evidence for safety or tolerability comprehensively. The current meta-analysis combined a sample of 3,622 patients from 14 short-term randomized, double-blind, placebo-controlled trials, fulfilling DSM-IV criteria for GAD[14]without other psychiatric or clinically serious medical conditions. Furthermore, it demonstrated the superior efficacy and well-tolerability of venlafaxine XR.

Venlafaxine XR has been approved by FDA and proved effective for the treatment of GAD[6,29], and mean change on HAM-A total scores from baseline to endpoint(MD = 3.31, 95%CI 1.44–5.18, P = 0.0005), response rate(OR = 1.83, 95%CI 1.58–2.12, P<0.00001), as well as remission (OR = 2.55, 95%CI 1.36–4.78, P = 0.003) provided further evidence on the therapeutic benefit of venlafaxine XR.

Tolerability was measured by discontinuation rate due to all-cause, AEs, lack of efficacy[30], and common TEAEs. The results indicated that patients treated with venlafaxine XR were more likely to discontinue the treatment due to AEs compared with placebo-treated patients (OR = 2.80) and discontinuation owing to inefficacy in patients receiving placebo was higher than venlafaxine XR(OR = 0.26). Moreover, the most frequent TEAEs were nausea, dry mouth, dizziness, insomnia,somnolence, and headache. The incidence of nausea, dry mouth, dizziness, insomnia and somnolence was more higher in venlafaxine XR than placebo while headache was not statistically different between the two groups. It should be noted that blood pressure had no significant changes in patients treated with venlafaxine XR(75-225mg/day), which was inconsistent with previous opinion that venlafaxine promoted elevation in blood pressure. The meta-analysis of these pooled data confirmed a statistically short-term effectiveness and well-tolerability.

The major advantage of our meta-analysis was the selection of all multicentered, randomized, double-blind, placebo-controlled trials. In addition to articles presented in the electronic databases, further articles were not ignored by reviewing the reference lists of published reports. Furthermore, we set strict inclusion criteria and owned large sample size.

The current meta-analysis had several limitations that should be noted. First, no restriction on fixed or flexible dose may increase heterogeneity and some results showed heterogeneity. We attempted to overcome such limitation by sensitivity analysis and showed the same results. Second, several studies were excluded in the analysis as a result of failure to extract data, e.g., graphs without illustration. Third, some potential bias (e.g. selection and reporting bias) were unclear or high. Given the limitations above,future surveys are warranted to generate more data to assess its cost-effectiveness[31] and relapse at follow-up.

In conclusion, our meta-analysis showed that venlafaxine XR (75–225 mg/day) is an effective pharmacological treatment option in efficacy and well-tolerability for adult patients with GAD. However, we should be cautious with the large dosage of venlafaxine XR in clinical practice. To provide more evidence of venlafaxine XR treatment, more high quality studies need to be conducted to explore the cost effectiveness of venlafaxine XR and warrant its effectiveness in children and adolescents with GAD.

## Supporting information

S1 File PRISMA 2009 checklist. (DOC)

S2 File Study protocol. (DOC)
