# Supplementary material for: Short-term efficacy and tolerability of venlafaxine extended release in adults with generalized anxiety disorder without depression: A meta-analysis
Source: PLoS One. 2017 Oct 5;12(10):e0185865. doi: 10.1371/journal.pone.0185865 (PMC5628888; doi:10.1371/journal.pone.0185865)
Supplement: S2 File — (DOC) [file pone.0185865.s002.doc]

**Short-term efficacy and tolerability of venlafaxine extended release in adults with generalized anxiety disorder without depression: A meta-analysis protocol**

Xinyuan Li, Lijun Zhu, Yingying Su,Shaokuan Fang

**Review question(s)**

Although efficacy of venlafaxine extended release (XR) for generalized anxiety disorder(GAD) has been reported in previous analyses in 2002 and 2004, the sample size was rather small and estimate of safety or tolerability was not clearly. The present analysis had the advantage of large sample size and provided evidence for tolerability.

**Searches**

Literature databases were searched, including Pubmed, Embase, Cochrane Central Register of Controlled Trials, Web of science and clinical trials.

Contact of experts: we attempt to contact the authors of relevant articles when needed. There were no restrictions related to the language or date of publication.

Types of study to be included

Types of study designs: experimental (randomized controlled trials).

**Participants/ population**

Patients aged or older than 18 years meeting the DSM-IV criteria for GAD

**Exposure(s)**

Venlafaxine XR or venlafaxine XR plus antipsychotics

**Comparator(s)/ control**

Placebo-controlled

**Outcome(s)**

The primary efficacy parameter was the mean change in HAM-A total scores from baseline to endpoint. The secondary efficacy parameters were the response and remission rates.

The primary tolerability of venlafaxine XR was evaluated by the discontinuation rates due to any reason ,AEs, and lack of efficacy. The second tolerability parameter was the incidence of the most frequently TEAEs.

**Data extraction, (selection and coding)**

Selection process:

Title and abstract screening: Teams of two reviewers will use the above eligibility criteria to screen titles and abstracts of identified citations in duplicate and independently for potential eligibility. We will get the full text for citations judged as potentially eligible by at least one of the two reviewers.

Full-text screening: Teams of two reviewers will use the above eligibility criteria to screen the full texts in duplicate and independently for eligibility. The teams of two reviewers will resolve disagreement by discussion or with the help of a third reviewer.

We will use standardized and pilot tested screening forms. We will conduct calibration exercises to ensure the validity of the selection process.

Data abstraction process:

Teams of two reviewers will abstract data from eligible studies in duplicate and independently. They will resolve disagreements by discussion or with the help of a third reviewer.

We will collect the following data: Age, sex distribution, number of enrolled participants, study-level inclusion and exclusion criteria, intervention details, treatment duration, reported outcomes, efficacy measures, and measure times.

We will use standardized and pilot tested data abstraction forms

We will conduct calibration exercises to ensure the validity of the data abstraction process.

**Risk of bias (quality) assessment:**

Teams of two reviewers will assess the risk of bias in each study in duplicate and independently. They will resolve disagreements by discussion or with the help of a third reviewer.

We will use the Cochrane Risk of Bias tool to assess the risk of bias in randomized trials.

We will calculate the risk of bias using the following criteria:

The likelihood of risk of bias included the selection bias (random sequence generation, allocation concealment), detection bias (blinding of outcome assessors, participant/personnel), reporting bias (selective reporting), and attrition bias (incomplete outcome data).

We will grade each potential source of bias as high, low or unclear risk of bias. We will use unclear when the authors did not report enough information for us to make the judgment.

We will not exclude any study based on quality.

**Strategy for data synthesis**

We will conduct a meta-analysis to pool the results across studies for venlafaxine XR as the exposure of interest, and ‘efficacy and tolerability’ as the outcome of interest.

The significance of the pooled estimates was determined by Z statistic, a statistical significance was set at a two-tailed P<0.05.

We will carry out statistical analysis using RevMan(version 5.3). For Dichotomous data, we will calculate the ORs for each study. For continuous data, we will calculate the mean difference for each study.

We will test the results for homogeneity using the I^2^ test and considered heterogeneity present if I^2^≥50% and P<0.05. If substantial heterogeneity was identified, the sensitivity analysis was performed.

The publication bias was assessed by the funnel plot and the Begg’s/Egger’s test using Stata Version 12.0 software.

**Dissemination plans**

We will publish results in international, peer-reviewed journals.
